# Postoperative Complications After Lung Cancer Resection in Patients with a Previous History of Head and Neck Cancer

**DOI:** 10.3390/cancers18172849

**Published:** 2026-09-03

**Authors:** Farzin Falahat-Noushzady, Sonia Herrero-Álvarez, Elisa M. Molanes-López, Roy Camacho-Leone, Carlos Alfredo Fraile-Olivero, Joaquín Calatayud-Gastardi, José Ramón Jarabo-Sarceda, Florentino Hernando-Trancho, Elena María Vara, Ana Maria Gómez-Martínez

**Affiliations:** 1 Department of Surgery, School of Medicine, Complutense University of Madrid, Avda. Complutense, S/N, 28040 Madrid, Spain; 2 Biostatistics Unit, Department of Statistics and Operations Research, School of Medicine, Complutense University of Madrid, Avda. Complutense, S/N, 28040 Madrid, Spain; 3 Department of Biochemistry and Molecular Biology, School of Medicine, Complutense University of Madrid, Avda. Complutense, S/N, 28040 Madrid, Spain

**Keywords:** head and neck cancer, lung cancer, second primary lung cancer, lung surgery complications, bronchopleural fistula

## Abstract

**Background/Objectives**: Surgical pulmonary resection in lung cancer remains the cornerstone of curative treatment; however, it is associated with considerable postoperative pulmonary complications. Patients previously treated for head and neck cancer (HNC) have a significantly increased risk of developing second primary lung cancer (LC) and a higher incidence of adverse pulmonary outcomes after LC resective surgery due to increased baseline frailty. **Methods**: A retrospective observational longitudinal study was conducted on 1679 patients who underwent surgical resection with curative intent for LC at the Thoracic Surgery Department of Hospital Clínico San Carlos, Madrid, Spain, between December 1989 and December 2024. Patients were divided into two groups based on the presence or absence of prior HNC: 87 with HNC vs. 1592 without. Special attention was paid to respiratory complications, particularly bronchopleural fistula (BPF), empyema, atelectasis, and pneumonia. Variables significantly associated with BPF and HNC in univariate analyses (excluding those related to other complications to avoid collinearity), as well as clinically relevant baseline differences between groups, were included in a stepwise multivariable logistic regression modeling procedure to identify independent risk factors associated with BPF. **Results**: In comparison with the control group, significantly higher rates of postoperative BPF (12.6% vs. 3.6%), empyema (10.3% vs. 2.8%), atelectasis (14.9% vs. 8.3%) and need of reintervention (10.3% vs. 4.2%) were found in the HNC group under unadjusted approach and stratified by surgery period. Sex, history of HNC, chronic obstructive pulmonary disease (COPD) and length of preoperative hospital stay were independently associated with BPF according to several multivariable logistic regression fits, which showed a moderate discriminatory capacity (AUC values ranging from 0.723 to 0.732). In particular, the presence of HNC was significantly associated with BPF, with adjusted odds ratio (aOR) estimates ranging from 3.562 (95% CI: 1.773 to 7.156) to 3.789 (95% CI: 1.863 to 7.709). **Conclusions**: In our study, COPD, male sex, preoperative hospital stay as well as HNC history were found independently associated with an increased odds of BPF after surgical treatment for LC. In particular, patients previously treated for HNC had more than threefold higher odds of developing BPF than those without a history of HNC, with this association remaining consistent over the 35-year study period.

## Simple Summary

The incidence of second primary cancers has increased in the past decade. It is not uncommon that head and neck cancer (HNC) patients develop second primary lung cancer (LC) due to a shared risk factor: tobacco smoking. Patients with HNC share common characteristics which predispose them to severe adverse respiratory outcomes after lung surgery. The objective of this study is to draw a comparison between the postoperative complications of patients diagnosed with LC who underwent radical surgical treatment and those who underwent the same treatment while having a personal history of HNC. The hypothesis of the researchers is that patients diagnosed with LC have an increased rate of postoperative complications if they also have a previous history of primary HNC, especially respiratory complications.

## 1. Introduction

Lung cancer (LC) remains the leading cause of cancer-related mortality worldwide despite major advances in screening strategies, systemic therapies, and surgical techniques, being responsible for more than 20% of annual cancer-related deaths. In terms of incidence, it ranks second after prostate cancer in men and breast cancer in women, respectively [1,2]. Surgical resection continues to represent the cornerstone of curative treatment for early-stage lung cancer and selected cases of locally advanced disease [2,3,4]. However, pulmonary resection is associated with considerable postoperative morbidity, with the incidence of general postoperative adverse events ranging from 9% to 53.4% [4].

Specifically, postoperative pulmonary complications (PPCs) occur in nearly 40% of patients, especially respiratory complications such as pneumonia, atelectasis, respiratory failure, prolonged air leak, and bronchial stump-related complications [2]. Several factors have been associated with increased risk of PPCs after lung cancer resection, including advanced age, sex, smoking history, chronic obstructive pulmonary disease (COPD), cardiovascular comorbidities, diabetes, impaired nutritional status, and reduced pulmonary reserve [2,5]. In recent years, attention has progressively shifted toward identifying particularly fragile surgical populations with a higher perioperative risk profile [2,5].

The number of cancer survivor patients has increased in the last decades due to advancements in early detection and treatment, leading to improved survival [6]. Longer survival rates result in an increased risk of developing second primary malignancies [7]. Among these populations, patients with previous head and neck cancer (HNC) represent a unique clinical group. Some series have reported a significant improvement in the five-year relative survival of HNC during the last two decades and showed a 15-year relative survival of 30% for men and 40% for women [8]. Other studies show that the 5-year overall survival rate for localized head and neck cancer ranges between 70% and 90%, while these rates are 25% to 60% in locoregionally advanced disease [9].

Numerous studies have identified smoking as a shared risk factor for head and neck and lung cancers [10,11,12]. Furthermore, research shows that patients with smoking-related cancers had a 51% higher risk of second primary malignancies, with head and neck cancer patients showing the greatest risk [12], as 70% to 80% of HNC is associated with a history of tobacco consumption [13]. Consequently, patients who survive HNC have a significantly increased risk of developing second primary lung cancer [14,15]. Beyond the well-recognized clinical association between HNC and second primary lung cancer, recent molecular studies have further highlighted the complex biological pathways involved in HNC progression and treatment response, emphasizing the heterogeneity of these tumors and the need for individualized therapeutic strategies [16].

As survival after HNC continues to improve, the management of second primary lung cancer in these patients has become an increasingly relevant clinical challenge. Compared with the general population, patients with HNC experience a higher incidence of adverse respiratory outcomes, as consistently reported across multiple studies [17]. In addition to their oncological burden, HNC patients frequently present multiple conditions that may predispose them to postoperative complications after lung surgery, such as previous cervical surgery, chemotherapy and radiotherapy, swallowing dysfunction, malnutrition, sarcopenia, smoking-related vasculopathy, and chronic alcohol consumption [5,14,17,18]. Studies show that smoking is a risk factor for PPCs and that patients undergoing neck surgery have significantly higher risk (2.3 times) of postoperative pneumonia compared to other surgeries, making HNC patients an especially vulnerable population [5].

The primary objective of the present study was to evaluate whether patients undergoing radical surgical treatment for LC with previous HNC present a higher prevalence of postoperative complications compared with patients with LC and no previous history of HNC. Special attention was directed toward respiratory complications and bronchopleural fistula (BPF) development given the particular frailty of this surgical population.

Although previous studies have reported increased postoperative morbidity after pulmonary resection in patients with previous HNC, most available series include relatively small cohorts and mainly evaluate overall postoperative complications. Data specifically addressing bronchopleural fistula and its associated risk factors remain scarce. Therefore, the present study aims to further characterize postoperative outcomes in one of the largest single-center cohorts reported to date.

## 2. Materials and Methods

A retrospective observational longitudinal study was performed including all consecutive patients who underwent curative-intent surgical resection for LC at the Thoracic Surgery Department of Hospital Clínico San Carlos (HCSC), Madrid, Spain, between December 1989 and December 2024. Within this cohort, patients diagnosed with HNC were identified. This study was based on data retrieved from a prospectively maintained institutional thoracic surgery database, in which demographic, clinical, surgical, pathological and postoperative variables are systematically recorded for all patients undergoing thoracic surgical procedures. The present investigation represents a retrospective analysis of these prospectively collected data.

Hospital Clínico San Carlos is a tertiary teaching hospital affiliated with the Complutense University of Madrid and a referral center for thoracic surgery in Madrid. The Department of Thoracic Surgery performs approximately 350–400 major thoracic surgical procedures annually, including anatomical lung resections for primary lung cancer. All patients with suspected or confirmed primary lung cancer are evaluated before treatment by a dedicated multidisciplinary Thoracic Oncology Tumor Board. This board includes thoracic surgeons, pneumologists, medical oncologists, radiation oncologists, radiologists, pathologists, rehabilitation physicians and specialized oncology nurses, with additional specialists participating whenever required. Clinical history, imaging studies, pathological findings, pulmonary function, performance status and comorbidities are systematically reviewed, and treatment recommendations are established by multidisciplinary consensus in accordance with current international guidelines. Surgical indications are therefore determined within a multidisciplinary framework, ensuring an individualized and evidence-based therapeutic approach for every patient.

Although surgical techniques and perioperative management evolved throughout the study period (1989–2024), patient selection and therapeutic decision-making were consistently based on multidisciplinary evaluation according to the standards of each period. Whenever indicated, particularly after right pneumonectomy, the bronchial stump was routinely reinforced with a vascularized flap using pericardial fat, parietal pleura or an intercostal muscle according to surgeon preference and intraoperative findings.

Inclusion criteria:

1. Patients diagnosed with LC who consecutively underwent lung surgery at HCSC.
2. Patients who received surgical treatment during the study period from 1989 to 2024.
3. Patients with LC and additional diagnosis of HNC treated with curative intent.
4. Patients who underwent surgical treatment of their LC with curative intention.
5. Patients with complete preoperative and postoperative clinical data available.

Exclusion criteria:

1. Patients with LC subjected to staging surgery.
2. Patients with LC who underwent diagnostic surgery (biopsy).
3. Patients with LC receiving palliative surgery.
4. Patients with LC who underwent exploratory thoracotomy due to unresectable disease.
5. Patients with LC who received neoadjuvant chemotherapy or radiotherapy for lung cancer
6. Patients with LC diagnosed with a malignancy other than HNC.

Demographic, clinical, oncological, and postoperative variables were retrospectively collected from a prospectively maintained institutional database. The primary outcome was the occurrence of postoperative complications after radical lung surgery. Each complication was recorded as a separate clinical outcome according to predefined diagnostic criteria, regardless of its severity. Since the objective of this study was to analyze the incidence and predictors of specific postoperative complications, rather than to assess overall postoperative morbidity, complications were analyzed individually and were not classified according to the Clavien–Dindo grading system.

The study covariates were: age (expressed in years at the date of surgery), gender (male, female), smoking habit, COPD, vasculopathy, diabetes mellitus, arterial hypertension, arrhythmia, valvulopathy, tuberculosis history, previous acute myocardial infarction, preoperative hospital stay, lung cancer staging, lung tumor location, resection type, overall postoperative complications, respiratory complications, pneumonia, bronchopleural fistula (BPF), empyema, atelectasis, hemothorax, pulmonary thromboembolism, chylothorax, reintervention, operative mortality, and postoperative mortality (defined as death occurring within 30 days after surgery). Special attention was directed toward respiratory complications, particularly bronchopleural fistula (BPF, defined as pathological communication involving the bronchial stump diagnosed clinically, radiologically, bronchoscopically, or intraoperatively), empyema, atelectasis, and pneumonia. Secondary outcomes included the identification of factors independently associated with BPF development. The data related to previous HNC included tumor site, histologic type, surgical procedure, and associated treatments (surgery, radiotherapy, chemotherapy).

Quantitative variables were expressed as mean and standard deviation (SD), as well as median and interquartile range (IQR) when appropriate. Qualitative variables were expressed as frequencies and percentages.

Comparisons between groups were performed using the Mann–Whitney U test for quantitative variables and the Chi-square test or Fisher’s exact test for qualitative variables, as appropriate. Given that this study spans more than three decades (1989–2024), a sensitivity analysis stratifying by the temporal period of surgery (1989–1999, 2000–2009, 2010–2019 and 2020–2024) was carried out in order to control for time-dependent confounding factors, such as historical shifts in surgical techniques, changes in perioperative care, anesthesia, or enhanced recovery protocols and evolving hospital guidelines over the eras. Under this approach, comparisons were performed using the likelihood ratio test corresponding to the univariate conditional logistic model stratified by the period of surgery, with the group variable as the outcome and the corresponding quantitative or qualitative variable as the explanatory variable. Conditional logistic regression allows data to be matched by the period of surgery, eliminating its confounding effect.

Subsequently, univariate analyses were performed to identify factors associated with bronchopleural fistula development in the overall study population, using both approaches (unadjusted and stratified by the period of surgery).

Variables showing statistical significance in univariate analyses, as well as clinically relevant baseline differences between groups, were included in multivariable logistic regression model to identify independent risk factors associated with BPF.

Variables directly representing postoperative complications related to BPF development were excluded from the multivariable model to avoid collinearity. To efficiently identify a parsimonious subset of predictors that balances predictive accuracy with model complexity, a bidirectional stepwise selection procedure was used for the complete-case multivariable analysis. From the final multivariable logistic regression model derived through this selection procedure, sensitivity analyses were conducted across several effective sample sizes, including one based on imputed data.

For each multivariable logistic regression model, several diagnostic analyses were performed, including calibration based on goodness-of-fit testing, multicollinearity assessment, residual analysis, and internal validation.

Statistical significance was established at *p*-value < 0.05. Statistical analyses were performed using JASP software (Version 0.98; [19]).

## 3. Results

**Figure 1:**
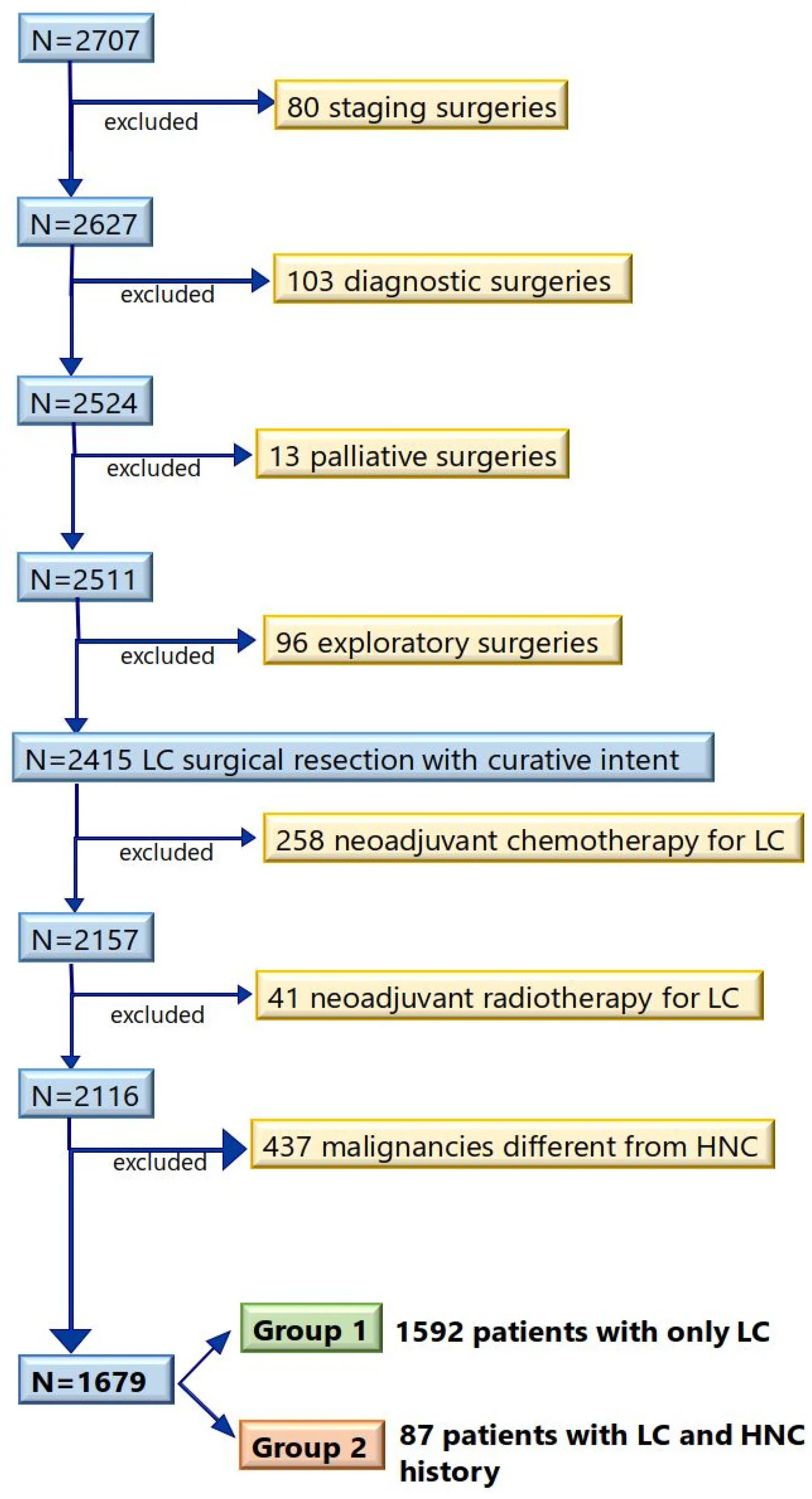
Patients included in this study after the inclusion and exclusion criteria were applied.

After the application of inclusion and exclusion criteria, two study groups were defined. From an initial cohort of 2707 patients with lung cancer diagnosis who received lung surgery, 80 were excluded due to staging surgery, 103 for being subject to diagnostic surgery, 13 for receiving palliative surgery, and 96 for undergoing exploratory thoracotomy. From the remaining cohort of 2415 patients who underwent surgical treatment of their LC with curative intention, 258 received neoadjuvant chemotherapy and 41 received neoadjuvant radiotherapy for LC. Of our resulting sample of 2116 patients, 437 were excluded from this study for presenting other second primary malignancies different from head and neck cancer. The final patient sample consisted of 1679 patients who were divided into two groups according to the presence or absence of previous HNC (Figure 1):

− Group 1 (control): A total of 1592 patients with isolated LC.
− Group 2: A total of 87 patients with LC and previous history of HNC.

### 3.1. Preoperative Characteristics

The number of patients included in every time period; lung cancer location, histological type and resection type; HNC location, histological type and treatment; and the time interval from HNC to LC are shown in Table A1, Table A2, Table A3, Table A4, Table A5, Table A6 and Table A7 of Appendix A.

The mean age of the overall cohort was similar between groups, with no statistically significant differences observed. Patients in the control group presented a mean age of 65.13 years (SD 9.74), whereas patients with previous HNC presented a mean age of 65.78 years (SD 6.46) (*p* = 0.911). The preoperative characteristics and comorbidities of Group 2 patients were similar to those observed in the control group in most of the variables studied, with no statistically significant differences except for the distribution of sex (with only 6.9% of females in Group 2 versus 20.2% in Group 1), vasculopathy (with a prevalence of 21.8% in Group 2 versus only 11.9% in Group 1), smoking habit (with only 5.8% of non-smokers in Group 2 versus 21.3% in Group 1), and lung cancer staging and resection type (Table 1). Stratification by the period of surgery revealed new statistically significant differences, particularly in the prevalence of arterial hypertension and diabetes mellitus. Also, there was a significant increase in the percentage of female patients and ex-smokers in both groups during each period of this study.

### 3.2. Postoperative Outcomes

In Table 2, the postoperative complications experienced after radical lung surgery are listed and compared between the two groups of interest. In comparison with the control group, significantly higher rates of postoperative bronchopleural fistula, empyema, atelectasis and need of reintervention were found in the HNC group under both approaches (unadjusted and stratified by period).

### 3.3. Factors Associated with Bronchopleural Fistula

Risk factors for bronchopleural fistula (BPF) in the entire target population (that is, considering both groups, Group 1 and Group 2, together) were investigated. Univariate analyses identified the following significant risk factors for fistula: male sex, COPD, smoking habit, preoperative stay, local infection, pneumonia, empyema and hemothorax (see Table 3). In addition, period-stratified analysis revealed statistically significant differences in arrhythmia distribution. However, tumor location, LC stage and resection type did not show statistically significant differences in BPF rate.

Multivariable logistic regression analyses revealed that sex, HNC history, preoperative stay, and COPD were independent risk factors for developing BPF when using a stepwise procedure based on those factors identified as significantly associated with fistula and HNC in the univariate analyses previously shown in Table 3 for fistula and in Table 1 and Table 2 for HNC, excluding those related to other complications (see Table 4). Specifically, the bidirectional stepwise procedure, initiated from the identified risk factors (sex, COPD, arterial hypertension, vasculopathy, arrhythmia, diabetes mellitus, smoking habit, lung cancer staging, preoperative stay, resection type, and HNC), added variables sequentially in four forward steps: sex, followed by HNC, preoperative stay, and COPD. The presence of HNC was statistically significant in the final multivariable logistic regression fit, with an adjusted odds ratio (aOR) estimate of 3.654 (95% CI from 1.810 to 7.374). This final fit (with an effective sample size of n = 1530, due to listwise removal of 149 patients with missing information in some of the variables involved in the stepwise procedure) yields an optimistic Area Under the Receiver Operating Characteristic (ROC) curve (AUC) of 0.732 (95% CI: 0.674 to 0.791), indicating a moderate discriminatory capacity to predict BPF. The primary model presented in Table 4 (complete-case analysis) was employed as the basis for subsequent comprehensive sensitivity analyses using different effective sample sizes and including exploratory variables not identified through the stepwise procedure.

Table 5 and Table 6 collect the same fit presented in Table 4 but with a larger effective sample size in each (n = 1606 in the fit of Table 5, after removing only 73 patients with missing values in preoperative stay, and n = 1679 in the fit of Table 6, after replacing those missing values with the median preoperative stay). The results shown in Table 5 and Table 6 are very similar to those presented in Table 4. Furthermore, similar results were obtained when period of surgery was included in each of the previous models, although it was not statistically significant (see, for instance, Table 7, where the results of the model presented in Table 4 with an effective sample size of n = 1530, now with period of surgery included, are displayed.). Therefore, controlling for the period of surgery, the previous findings are robust.

The classic Hosmer–Lemeshow test (configured with an initial target of g = 10 risk groups) demonstrated adequate calibration, that is, adequate agreement between observed and expected event frequencies, ruling out a significant lack of fit (${Χ}_{6}^{2}$ = 7.900, *p*-value = 0.246 > 0.05 for model in Table 4; ${Χ}_{6}^{2}$ = 5.623, *p*-value = 0.467 > 0.05 for model in Table 5; ${Χ}_{6}^{2}$ = 5.048, *p*-value = 0.538 > 0.05 for model in Table 6; ${Χ}_{8}^{2}$ = 7.062, *p*-value = 0.530 > 0.05 for model in Table 7; and ${Χ}_{8}^{2}$ = 5.046, *p*-value = 0.753 > 0.05 for model in Table 8). Note that due to tied predicted probabilities among identical predictor profiles, the risk groups were collapsed dynamically by the software in some models, resulting in fewer than eight degrees of freedom (df = g − 2) in the corresponding chi-squared statistics.

To evaluate multicollinearity, Variance Inflation Factors (VIFs) were computed and displayed in the last column of Table 4, Table 5, Table 6, Table 7 and Table 8. All predictors showed negligible collinearity, with all VIF values falling well below the conservative threshold of 5.0.

Residual analysis detected only two outliers (patients with standardized residuals >3 or <−3) across all four multivariable models. Regression diagnostics were subsequently performed using DFBETAS, a metric that measures, for each predictor variable, the standardized change in its regression coefficient when a specific observation is excluded, to evaluate whether these outliers, or any other individual data points, represented influential observations. Given the large effective sample sizes (n = 1530, 1606, 1679, 1530 and 1530, respectively), the size-adjusted threshold recommended by Belsley et al. (1980) of ±2n (0.0511, 0.0499, 0.0488, 0.0511 and 0.0511) was applied. Only the two outliers (approximately 0.13% of the different effective sample sizes) exceeded this strict criterion for the sex predictor (with values that ranged between 0.251 and 0.322 across the five models, well below the conservative absolute threshold of 1.0.). For all other observations and predictors, DFBETAS values remained remarkably low.

Internal validation via 10,000 bootstrap resamples yielded bias-corrected AUC indexes with values very close to the optimistic AUC values computed from the original sample. Similarly, corresponding 95% confidence intervals yielded very similar findings to those previously reported from the original sample. Below, we present the bias-corrected AUC indexes for each model, with their corresponding 95% CI: bias-corrected AUC = 0.725 (95% CI: 0.671 to 0.788) for the model of Table 4; bias-corrected AUC = 0.716 (95% CI: 0.663 to 0.779) for the model of Table 5; bias-corrected AUC = 0.722 (95% CI: 0.671 to 0.784) for the model of Table 6; bias-corrected AUC = 0.703 (95% CI: 0.665–0.790) for the model of Table 7; and bias-corrected AUC = 0.724 (95% CI: 0.686–0.802) for the model of Table 8.

## 4. Discussion

Developing a second primary cancer may be closely related to the first primary tumor and can be influenced by different factors such as prior oncological treatments (surgery, radiotherapy, chemotherapy), shared genetic mutations between first and second primary cancers and exposure to certain carcinogenic substances [20]. Several studies have demonstrated a strong correlation between primary HNC and the development of second primary lung cancer, with a prevalence ranging from 5% to 19% [21]. Wang et al. in their systematic review and meta-analysis of 9 million cancer patients showed that survivors of primary HNC developed second primary lung cancer more frequently [20]. Moreover, Tanjak et al. reported a significant association between HNC history and developing a second primary lung cancer (RR 2.41; 95% CI: 1.58–3.58) [22]. In survivors of HNC, second primary lung cancer frequently develops due to a main shared risk factor, which is tobacco smoking [10,23]. It is a remarkable finding in the present study that there was a significantly lower proportion of non-smokers (5.8% vs. 21.4%) and a significantly higher proportion of ex-smokers (55.8% vs. 31.6%) in the HNC group vs. the no-HNC group, indicating that the harmful impact of smoking remains present even after discontinuing the smoking habit, leading to a second malignancy development. Similar findings were reported in other studies such as Shiels et al. [24], observing an increased risk of developing a second cancer in patients who were active smokers before first cancer diagnosis.

The present study may suggest that patients who survive HNC and subsequently develop second primary lung cancer represent a highly vulnerable population with unique characteristics that predispose them to an increased risk of postoperative pulmonary complications after surgical treatment of LC. These findings are consistent with the previous literature describing increased postoperative complications in patients with head and neck malignancies undergoing pulmonary resection. Benz BG et al. reported 97% pulmonary complications after lung surgery in a group of HNC patients who underwent pulmonary resection, with 34% major complications [25]. Similar results were found by Briend et al. [13] in their case-control study comparing a control group of patients undergoing lung resection (LR) for lung cancer without previous HNC vs. a group of patients undergoing LR after previous HNC. A significantly higher rate of major complications occurred in the HNC patient group (42% [95% CI, 29.4–55.4%]) compared to the non-HNC group (16% [95% CI, 9.2–22.5%]), postoperative pneumonia being the most frequent complication (32% in the HNC group vs. 10% in the control group). In their study, HNC was found to be a major independent risk factor for serious postoperative complications after lung cancer surgery.

Similarly, Herrera et al.’s group [26] demonstrated a significantly increased risk of aspiration pneumonia and pulmonary complications after lung cancer surgery in patients with previous HNC compared to a control group of patients that underwent lung cancer surgery but did not present HNC history. They also observed a significantly higher incidence of bronchopleural fistula in the HNC group (3.9%) vs. the control group (0.8%). In contrast with Herrera et al., our group did not find a significantly higher rate of postoperative pneumonia in the HNC group vs. the no-HNC group, but in accordance with the mentioned study, our group also observed a higher incidence of bronchopleural fistula in the HNC group, even when stratifying by surgery period.

No statistically significant differences were observed between our two study groups when comparing overall postoperative complications or respiratory complications both in the unadjusted analysis and stratified by periods. However, analysis of individual complications revealed that patients with previous HNC undergoing lung cancer surgery had a significantly higher risk of developing atelectasis (14.9 vs. 8.3%), bronchopleural fistula (12.6% vs. 3.6%), empyema (10.3% vs. 2.9%), and need for reintervention (10.3% vs. 4.1%) than patients without HNC history. These findings could suggest that a history of HNC may predispose patients to specific pulmonary postoperative complications following lung resection, as the risk remains significantly higher in the HNC group throughout the four periods of this study for the complications mentioned above (atelectasis, BPF, empyema and need for reintervention). Although in our series atelectasis was the most frequent complication, bronchopleural fistula showed the strongest statistical association (*p* < 0.001) with HNC history, emerging as one of the most clinically relevant complications due to its strong association with local infection (11.8% vs. 2.9%), pneumonia (22.1 vs. 8.3%), empyema (38.2% vs. 1.7%), hemothorax (10.3% vs. 2.7%), reintervention (48.5% vs. 2.7%), and mortality (11.8% vs. 3.2) after further in-depth analysis.

There was a need to study the factors associated with bronchopleural fistula in the entire target population of patients given that our study groups were not homogeneous: overall, patients with previous HNC demonstrated a demographic and clinical profile characterized by male predominance, heavier tobacco exposure and a higher prevalence of smoking-related vasculopathy compared with patients with no previous history of HNC. Also, no stage IIIB or IV for lung cancer was found in the HNC group. As reported in the literature, there are some patient-related factors that cause healing impairment and increase the risk of developing postoperative BPF, such as advanced age, male gender, smoking, diabetes mellitus, COPD and poor nutritional status [27,28].

Regarding baseline patient characteristics and comorbidities in our cohort, the development of BPF was significantly higher in males (97.1% globally, 100% during 1st, 2nd and 3rd periods and 87.8% during 4th period), patients with COPD (54.4% globally, 20% during 1st period, 66.7% during 2nd and 3rd periods, 54.5% during 4th period) and heavy smokers (52.4% globally, 84.6% during 1st period, 50% during 2nd period, 47.1% during 3rd period, 27.3% during 4th period) followed by ex-smokers (34.9% globally, 6.3% during 1st period, 62.5% during 2nd period, 60% during 3rd period and 79.2% during 4th period). Therefore, some of the baseline factors and comorbidities already known to differentiate the HNC group from the non-HNC group (sex and smoking status) were also associated with BPF development. It is interesting to note that in the multivariable analysis, previous HNC was shown to be independently associated with BPF (OR = 3.654, 95% CI: 1.810–7.374; see Table 4). After adjusting for the effects of sex, COPD, and preoperative stay, previous HNC was associated with a 3.65-fold increase in the odds of experiencing BPF. Therefore, this association could not be explained by sex, COPD, and preoperative stay alone. Furthermore, given that the VIF values were low for all four predictors in the model, the isolated effect of each variable could be estimated with precision. This indicates that in our study in the target population of patients diagnosed with LC who underwent radical surgical treatment, the odds of experiencing bronchopleural fistula was more than three times higher for patients with previous HNC than for patients without HNC history. It is worth noting that these findings remained stable over the 35-year study period; adjusting for the period of surgery in the multivariable logistic regression model did not materially change the aORs of the primary predictors, and the period itself was not statistically significant. The consistency of these results throughout the whole study period could suggest that the observed associations were robust and were not substantially influenced by temporal changes in surgical practice or perioperative management. This observation correlates to the already mentioned Briend’s et al. study [14], as their group reported HNC as an independent risk factor for serious postoperative complications after lung cancer surgery. Also, COPD was found to be a condition that independently increased the risk of developing bronchopleural fistula following LC lung surgery in our series, in accordance with the literature [27,28].

One noteworthy finding of the present study was that, despite the recognized association of vasculopathy with impaired vascularization, tissue ischemia, and delayed wound healing [29], and of smoking with pulmonary dysfunction, impaired tissue healing, infection, respiratory complications, anastomotic leakage and systemic vascular disease [30,31], neither factor was independently associated with BPF in the final multivariable model after adjustment for other factors.

The association between head and neck cancer and postoperative bronchopleural fistula in our work is interesting, as studies have reported the relationship between low body mass index and the risk of postoperative BPF [32]. In the case of head and neck cancer survivors, the frequent adverse effects of their cancer treatment and the proximity to anatomical and functional structures responsible for food intake and swallowing may lead to reduced nutritional intake and malnutrition [33], which could make this group of patients more vulnerable to BPF complication following LC surgery. Nevertheless, our study did not collect detailed information regarding nutritional status, body mass index, serum albumin, sarcopenia, swallowing dysfunction, radiation dose or treatment-related toxicity, considering this fact a limitation. Consequently, these pathophysiological mechanisms should be considered biologically plausible hypotheses rather than causal explanations of our findings. Prospective studies incorporating these variables are needed to clarify the relative contribution of each factor to postoperative bronchial stump healing.

Also, there was a significantly higher reintervention rate (48.5% vs. 2.6%) and postoperative mortality (12% vs. 3%) amongst patients who showed BPF compared with those who did not show this complication. Similarly to our work, Fernandez et al. [34] found in their study that postoperative bronchopleural fistula after resection for primary lung cancer was associated with worse survival. Moreover, patients in the HNC group presented significantly higher rates of reintervention (10.3%) compared to the no-HNC group (4.1%), potentially suggesting that previous HNC predisposes to more severe complications after LC resection surgery.

The findings in the present study have several potential clinical implications. Patients previously treated for HNC should be considered a high-risk subgroup when being evaluated for lung resection. Recognition of this increased risk may facilitate more accurate preoperative counselling and perioperative risk stratification. These HNC patients may benefit from comprehensive preoperative optimization, including smoking cessation, nutritional assessment, pulmonary prehabilitation and, when appropriate, evaluation of swallowing function in order to optimize treatment outcomes, reduce postoperative complications, and ultimately improve long-term prognosis when undergoing lung cancer surgery [33,35]. In addition, the mentioned strategies complemented by psychosocial interventions aimed at reducing cancer-related distress may contribute to improved psychological well-being and quality of life [36].

From a surgical perspective, meticulous bronchial stump management and the selective use of vascularized bronchial stump reinforcement in high-risk procedures may contribute to reducing the risk of bronchopleural fistula in this specific population [28]. Although prospective studies are required to determine whether these strategies improve outcomes, our findings support a multidisciplinary and individualized perioperative approach for patients with previous HNC undergoing lung cancer surgery. Finally, in the context of emerging technologies in healthcare and the growing integration of artificial intelligence, future multicenter studies with larger cohorts could assess machine learning and explainable AI approaches to improve individualized prediction of BPF risk and support the development and external validation of clinically applicable predictive models [37].

### Limitations

An important limitation of this study is the prolonged inclusion period (1989–2024), during which thoracic surgery and oncology experienced profound changes. Throughout the study period, our institution progressively incorporated several technical advances including modern stapling devices, improvements in bronchial stump closure techniques, better perioperative anesthetic management and enhanced postoperative recovery protocols. Video-assisted thoracoscopic surgery was progressively introduced during the later years of this study. Owing to the retrospective nature of the database, these temporal changes could not be analyzed separately and therefore constitute another limitation of this study. Likewise, advances in molecular characterization of lung cancer, targeted therapies and immunotherapy have substantially modified patient selection and overall oncological management. While these developments may have influenced postoperative outcomes over time, the retrospective single-center design did not allow adjustment for all temporal changes. Although after adjustment for surgical period the estimates remained broadly consistent, residual temporal confounding cannot be excluded, as the analytical model may not fully capture all time-varying factors that could have influenced patient selection, perioperative management, or outcomes over the study period. Therefore, our findings should be interpreted considering the inherent temporal heterogeneity of this institutional cohort.

In addition, the small HNC group in the present work can be attributed to the study population being restricted to patients who ultimately underwent pulmonary resection. Therefore, patients considered medically inoperable because of poor pulmonary reserve, advanced disease or severe comorbidity were not represented. Consequently, a degree of selection bias and a potential healthy-survivor effect cannot be excluded.

## 5. Conclusions

The present study showed higher rates of pneumonia and local infections in the HNC group with no statistical differences with the non-HNC group. Patients with previous HNC undergoing surgical treatment for LC had a significantly higher incidence of atelectasis, bronchopleural fistula, empyema and reintervention rate in this cohort.

In our study, COPD, male sex, preoperative hospital stay as well as HNC history were found independently associated with an increased odds of bronchopleural fistula development after surgical treatment for LC. Lung tumor location, stage and lung resection type were not significantly associated with postoperative BPF in this study. Within our study population, the odds of experiencing BPF were more than three times higher for patients with previous HNC than for patients without HNC history, a finding that remained stable over the 35-year study period.

## Figures and Tables

**Table 1:** Characteristics of all included patients (n = 1679), by group. AMI: acute myocardial infarction. COPD: chronic obstructive pulmonary disease. OR: odds ratio. Statistically significant differences are shown in bold. *p*-value of the comparison between the two groups: *** *p* < 0.001; ** *p* < 0.01; * *p* < 0.05.

Variable	Group 1—No HNC (n = 1592)	Group 2—HNC (n = 87)	*p*-Value
**Age (years)**	65.13 (9.74) 66 (59–72) 1 missing	65.78 (6.46) 65 (62–70)	0.911
**Age stratified by period**			0.654
Sex ** **Male** **Female**	1270 (79.8%) 322 (20.2%)	81 (93.1%) 6 (6.9%)	**0.002** **OR = 0.29** **(0.13 to 0.68)**
Sex stratified by period ***			**<0.0001** **OR = 0.23** **(0.10 to 0.55)**
**1st period: Male** **Female**	284 (90.7%) 29 (9.3%)	16 (100%) 0 (0%)	
**2nd period: Male** **Female**	429 (90.7%) 44 (9.3%)	16 (100%) 0 (0%)	
**3rd period: Male** **Female**	319 (73.8%) 113 (26.2%)	29 (96.7%) 1 (3.3%)	
**4th period: Male** **Female**	238 (63.6%) 136 (36.4%)	20 (80%) 5 (20%)	
**Associated pathology** **No** **Yes**	255 (16.0%) 1337 (84.0%)	16 (18.4%) 71 (81.6%)	0.558
**Associated pathology stratified by period**			0.346
**COPD** **No** **Yes**	992 (62.3%) 600 (37.7%)	48 (55.2%) 39 (44.8%)	0.182
**COPD stratified by period**			0.218
**Tuberculosis** **No** **Yes**	1505 (94.5%) 87 (5.5%)	85 (97.7%) 2 (2.3%)	0.320
**Tuberculosis stratified by period**			0.161
**Arterial hypertension** **No** **Yes**	1090 (68.5%) 502 (31.5%)	67 (77.0%) 20 (23.0%)	0.094
Arterial hypertension stratified by period *			**0.014** **OR = 0.53** **(0.31 to 0.90)**
**1st period: No** **Yes**	289 (92.3%) 24 (7.7%)	15 (93.8%) 1 (6.2%)	
**2nd period: No** ** Yes**	372 (78.6%) 101 (21.4%)	13 (81.2%) 3 (18.8%)	
**3rd period: No** ** Yes**	249 (57.6%) 183 (42.4%)	21 (70%) 9 (30%)	
**4th period: No** ** Yes**	180 (48.1%) 194 (51.9%)	18 (72%) 7 (28%)	
Vasculopathy ** **No** **Yes**	1403 (88.1%) 189 (11.9%)	68 (78.2%) 19 (21.8%)	**0.006** **OR = 2.07** **(1.22 to 3.53)**
Vasculopathy stratified by period *			**0.015** **OR = 2.03** **(1.19 to 3.48)**
**1st period: No** **Yes**	300 (95.8%) 13 (4.2%)	14 (87.5%) 2 (12.5%)	
**2nd period: No** ** Yes**	413 (87.3%) 60 (12.7%)	13 (81.2%) 3 (18.8%)	
**3rd period: No** ** Yes**	367 (85.0%) 65 (15.0%)	25 (83.3%) 5 (16.7%)	
**4th period: No** ** Yes**	323 (86.4%) 51 (13.6%)	16 (64%) 9 (36%)	
**Valvulopathy** **No** **Yes**	1557 (97.8%) 35 (2.2%)	86 (98.9%) 1 (1.1%)	1.000
**Valvulopathy stratified by period**			0.501
**History of AMI** **No** **Yes**	1466 (92.1%) 126 (7.9%)	84 (96.6%) 3 (3.4%)	0.128
**History of AMI stratified by period**			0.094
**Arrhythmia** **No** **Yes**	1475 (92.7%) 117 (7.3%)	81 (93.1%) 6 (6.9%)	0.875
**Arrhythmia stratified by period**			0.733
**Diabetes mellitus** **No** **Yes**	1353 (85.0%) 239 (15.0%)	80 (92.0%) 7 (8.0%)	0.074
Diabetes mellitus stratified by period *			**0.030** **OR = 0.45** **(0.21 to 0.99)**
**1st period: No** **Yes**	294 (93.9%) 19 (6.1%)	16 (100%) 0	
**2nd period: No** ** Yes**	414 (87.5%) 59 (12.5%)	15 (93.8%) 1 (6.2%)	
**3rd period: No** ** Yes**	357 (82.6%) 75 (17.4%)	26 (86.7%) 4 (13.3%)	
**4th period: No** ** Yes**	288 (77%) 86 (23%)	23 (92%) 2 (8%)	
Smoking habit *** **No** **20 PY** **40 PY** **Ex**	314 (21.3%) 119 (8.1%) 582 (39.4%) 461 (31.2%) 116 missing	5 (5.8%) 3 (3.5%) 30 (34.9%) 48 (55.8%) 1 missing	**<0.0001**
Smoking habit stratified by period ***			**<0.0001**
**1st period: No** **20 PY** **40 PY** **Ex**	71 (28.4%) 36 (14.4%) 132 (52.8%) 11 (4.4%) 63 missing	2 (12.5%) 0 13 (81.2%) 1 (6.3%)	
**2nd period: No** ** 20 PY** ** 40 PY** ** Ex**	73 (15.8%) 50 (10.8%) 229 (49.5%) 111 (24.0%) 10 missing	2 (12.5%) 1 (6.2%) 3 (18.8%) 10 (62.5%)	
**3rd period: No** ** 20 PY** ** 40 PY** ** Ex**	103 (25.4%) 28 (6.9%) 129 (31.8%) 146 (36%) 26 missing	1 (3.3%) 2 (6.7%) 9 (30%) 18 (60%)	
**4th period: No** ** 20 PY** ** 40 PY** ** Ex**	67 (18.8%) 5 (1.4%) 92 (25.8%) 193 (54.1%) 17 missing	0 0 5 (20.8%) 19 (79.2%) 1 missing	
Lung cancer staging ** **0** **I** **II** **IIIA** **IIIB** **IV**	6 (0.4%) 780 (49.0%) 403 (25.3%) 320 (20.1%) 47 (3.0%) 36 (2.3%)	0 51 (58.6%) 9 (10.3%) 27 (31.0%) 0 0	**0.002**
Lung cancer staging stratified by period **			**0.003**
**1st period: 0** **I** **II** **IIIA** **IIIB** **IV**	0 107 (34.2%) 99 (31.6%) 92 (29.4%) 11 (3.5%) 4 (1.3%)	0 7 (43.8%) 3 (18.8%) 6 (37.5%) 0 0	
**2nd period: 0** ** I** ** II** ** IIIA** ** IIIB** ** IV**	0 203 (42.9%) 126 (26.6%) 110 (23.3%) 15 (3.2%) 19 (4.0%)	0 11 (68.8%) 1 (6.2%) 4 (25.0%) 0 0	
**3rd period: 0** ** I** ** II** ** IIIA** ** IIIB** ** IV**	3 (0.7%) 241 (55.8%) 86 (19.9%) 76 (17.6%) 19 (4.4%) 7 (1.6%)	0 16 (53.3%) 2 (6.7%) 12 (40.0%) 0 0	
**4th period: 0** ** I** ** II** ** IIIA** ** IIIB** ** IV**	3 (0.8%) 229 (61.2%) 92 (24.6%) 42 (11.2%) 2 (0.5%) 6 (1.6%)	0 17 (68%) 3 (12%) 5 (20%) 0 0	
**Tumor location** **Intermediate bronchus** **Right main bronchus** **Left main bronchus** **Right lower lobe** **Left lower lobe** **Right middle lobe** **Right upper lobe** **Left upper lobe**	38 (2.4%) 41 (2.6%) 75 (4.7%) 254 (15.9%) 223 (14%) 87 (5.5%) 472 (29.6%) 402 (25.3%)	2 (2.3%) 0 1 (1.1%) 16 (18.4%) 13 (14.9%) 4 (4.6%) 25 (28.7%) 26 (29.9%)	0.580
**Tumor location stratified by period**			0.504
**Preoperative stay (days)**	2.84 (5.24) 1 (1–1) 72 missing	2.20 (3.92) 1 (1–1) 1 missing	0.266
**Preoperative stay (days) stratified by period**			0.220
**Resection type**			**0.020**
**Bilobectomy** **Lobectomy** **Pneumonectomy: Right, Left** **Segmentectomy**	102 (6.4%) 1046 (65.7%) 116 (7.3%): 41, 75 328 (20.6%)	11 (12.6%) 60 (69.0%) 1 (1.1%): 0, 1 15 (17.2%)	
Resection type stratified by period *			**0.020**
**1st period: Bilobectomy** **Lobectomy** **Pneumonectomy: Right, Left** **Segmentectomy**	30 (9.6%) 190 (60.7%) 36 (11.5%): 10, 26 57 (18.2%)	2 (12.5%) 13 (81.2%) 0 1 (6.3%)	
**2nd period: Bilobectomy** ** Lobectomy** ** Pneumonectomy: Right, Left** ** Segmentectomy**	32 (6.8%) 290 (61.3%) 63 (13.3%): 30, 33 88 (18.6%)	2 (12.5%) 13 (81.2%) 0 1 (6.3%)	
**3rd period: Bilobectomy** ** Lobectomy** ** Pneumonectomy: Right, Left** ** Segmentectomy**	22 (5.1%) 312 (72.2%) 13 (3.0%): 1, 12 85 (19.7%)	3 (10.0%) 16 (53.3%) 1 (3.3%): 0, 1 10 (33.3%)	
**4th period: Bilobectomy** ** Lobectomy** ** Pneumonectomy: Right, Left** ** Segmentectomy**	18 (4.8%) 254 (67.9%) 4 (1.1%): 0, 4 98 (26.2%)	4 (16.0%) 18 (72.0%) 0 3 (12.0%)	

**Table 2:** Postoperative complications by group (n = 1679) AMI: acute myocardial infarction. BPF: bronchopleural fistula. OR: odds ratio. Statistically significant differences are shown in bold. *p*-value of the comparison between the two groups: *** *p*< 0.001; ** *p* < 0.01; * *p* < 0.05.

Variable	Group 1—No HNC (n = 1592)	Group 2—HNC (n = 87)	*p*-Value
**Complications** **No** **Yes**	926 (58.2%) 666 (41.8%)	47 (54.0%) 40 (46.0%)	0.446
**Complications stratified by period**			0.669
**Respiratory complications** **No** **Yes**	1286 (80.8%) 306 (19.2%)	65 (74.7%) 22 (25.3%)	0.165
**Respiratory complications stratified by period**			0.167
**Local infection** **No** **Yes**	1543 (96.9%) 49 (3.1%)	82 (94.3%) 5 (5.7%)	0.198
**Local infection stratified by period**			0.208
**Pneumonia** **No** **Yes**	1455 (91.4%) 137 (8.6%)	76 (87.4%) 11 (12.6%)	0.196
**Pneumonia stratified by period**			0.301
BPF *** **No** **Yes**	1535 (96.4%) 57 (3.6%)	76 (87.4%) 11 (12.6%)	**<0.001** **OR = 3.90** **(1.96 to 7.73)**
BPF stratified by period ***			**<0.001** **OR = 4.13** **(2.07 to 8.24)**
**1st period: No** **Yes**	300 (95.8%) 13 (4.2%)	14 (87.5%) 2 (12.5%)	
**2nd period: No** ** Yes**	453 (95.8%) 20 (4.2%)	12 (75%) 4 (25%)	
**3rd period: No** ** Yes**	419 (97%) 13 (3%)	25 (83.3%) 5 (16.7%)	
**4th period: No** ** Yes**	363 (97.1%) 11 (2.9%)	25 (100%) 0	
Empyema ** **No** **Yes**	1547 (97.2%) 45 (2.8%)	78 (89.7%) 9 (10.3%)	**0.001** **OR = 3.97** **(1.87 to 8.41)**
Empyema stratified by period **			**0.001** **OR = 4.32** **(2.01 to 9.27)**
**1st period: No** **Yes**	296 (94.6%) 17 (5.4%)	13 (81.2%) 3 (18.8%)	
**2nd period: No** ** Yes**	460 (97.3%) 13 (2.7%)	13 (81.2%) 3 (18.8%)	
**3rd period: No** ** Yes**	422 (97.7%) 10 (2.3%)	27 (90%) 3 (10%)	
**4th period: No** ** Yes**	369 (98.7%) 5 (1.3%)	25 (100%) 0	
Atelectasis * **No** **Yes**	1462 (91.7%) 130 (8.3%)	74 (85.1%) 13 (14.9%)	**0.028** **OR = 1.98** **(1.07 to 3.66)**
Atelectasis stratified by period *			**0.022** **OR = 2.20** **(1.18 to 4.12)**
**1st period: No** **Yes**	302 (96.5%) 11 (3.5%)	14 (87.5%) 2 (12.5%)	
**2nd period: No** ** Yes**	411 (86.9%) 62 (13.1%)	11 (68.8%) 5 (31.2%)	
**3rd period: No** ** Yes**	395 (91.4%) 37 (8.6%)	26 (86.7%) 4 (13.3%)	
**4th period: No** ** Yes**	354 (94.7%) 20 (5.3%)	23 (92%) 2 (8%)	
**Hemothorax** **No** **Yes**	1546 (97.1%) 46 (2.9%)	83 (95.4%) 4 (4.6%)	0.325
**Hemothorax stratified by period**			0.460
**Pulmonary thromboembolism** **No** **Yes**	1577 (99.1%) 15 (0.9%)	87 (100%) 0	1
**Pulmonary thromboembolism stratified by period**			0.185
**Perioperative AMI** **No** **Yes**	1577 (99.1%) 15 (0.9%)	87 (100%) 0	1
**Perioperative AMI stratified by period**			0.199
**Chylothorax** **No** **Yes**	1587 (99.7%) 5 (0.3%)	87 (100%) 0	1
**Chylothorax stratified by period**			0.176
Reintervention * **No** **Yes**	1525 (95.8%) 67 (4.2%)	78 (89.7%) 9 (10.3%)	**0.014** **OR = 2.63** **(1.26 to 5.46)**
Reintervention stratified by period *			**0.019** **OR = 2.65** **(1.27 to 5.56)**
**1st period: No** **Yes**	290 (92.7%) 23 (7.3%)	12 (75%) 4 (25%)	
**2nd period: No** ** Yes**	458 (96.8%) 15 (3.2%)	15 (93.8%) 1 (6.2%)	
**3rd period: No** **Yes**	412 (95.4%) 20 (4.6%)	26 (86.7%) 4 (13.3%)	
**4th period: No** **Yes**	365 (97.6%) 9 (2.4%)	25 (100%) 0	
**Operative mortality** **No** **Yes**	1591 (99.9%) 1 (0.1%)	87 (100%) 0	1
**Operative mortality stratified by period**			0.109
**Postoperative mortality** **No** **Yes**	1534 (96.4%) 58 (3.6%)	86 (98.9%) 1 (1.1%)	0.365
**Postoperative mortality stratified by period**			0.202

**Table 3:** Factors associated with bronchopleural fistula (BPF) in the entire target population of patients (n = 1679). AMI: acute myocardial infarction. COPD: chronic obstructive pulmonary disease. OR: odds ratio. Statistically significant differences are shown in bold. *p*-value of the comparison between the two groups: *** *p*< 0.001; ** *p* < 0.01; * *p* < 0.05.

Variable	Non BPF (n = 1611)	BPF (n = 68)	*p*-Value
**Age (years)**	65.11 (9.65) 66 (59–72) 1 missing	66.53 (8.12) 67 (62–73)	0.231
**Age (years) stratified by period**			0.165
Sex *** **Male** **Female**	1285 (79.8%) 326 (20.2%)	66 (97.1%) 2 (2.9%)	**<0.001** **OR = 0.12** **(0.03 to 0.49)**
Sex stratified by period ***			**<0.0001** **OR = 0.13** **(0.03 to 0.53)**
**1st period: Male** **Female**	285 (90.8%) 29 (9.2%)	15 (100%) 0	
**2nd period: Male** ** Female**	421 (90.5%) 44 (9.5%)	24 (100%) 0	
**3rd period: Male** ** Female**	330 (74.3%) 114 (25.7%)	18 (100%) 0	
**4th period: Male** ** Female**	249 (64.2%) 139 (35.8%)	9 (81.8%) 2 (18.2%)	
**Associated pathology** **No** **Yes**	259 (16.1%) 1352 (83.9%)	12 (17.6%) 56 (82.4%)	0.730
**Associated pathology by period**			0.999
COPD ** **No** **Yes**	1009 (62.6%) 602 (37.4%)	31 (45.6%) 37 (54.4%)	**0.005** **OR = 2.00** **(1.23 to 3.26)**
COPD stratified by period **			**0.003** **OR = 2.15** **(1.30 to 3.58)**
**1st period: No** **Yes**	262 (83.4%) 52 (16.6%)	12 (80%) 3 (20%)	
**2nd period: No** ** Yes**	277 (59.6%) 188 (40.4%)	8 (33.3%) 16 (66.7%)	
**3rd period: No** ** Yes**	234 (52.7%) 210 (47.3%)	6 (33.3%) 12 (66.7%)	
**4th period: No** ** Yes**	236 (60.8%) 152 (39.2%)	5 (45.5%) 6 (54.5%)	
**Tuberculosis** **No** **Yes**	1527 (94.8%) 84 (5.2%)	63 (92.6%) 5 (7.4%)	0.404
**Tuberculosis stratified by period**			0.482
**Arterial hypertension** **No** **Yes**	1106 (68.7%) 505 (31.3%)	51 (75.0%) 17 (25.0%)	0.268
**Arterial hypertension stratified by period**			0.504
**Vasculopathy** **No** **Yes**	1410 (87.5%) 201 (12.5%)	61 (89.7%) 7 (10.3%)	0.593
**Vasculopathy stratified by period**			0.650
**Valvulopathy** **No** **Yes**	1575 (97.8%) 36 (2.2%)	68 (100%) 0	0.398
**Valvulopathy stratified by period**			0.444
**History of AMI** **No** **Yes**	1485 (92.2%) 126 (7.8%)	65 (95.6%) 3 (4.4%)	0.301
**History of AMI stratified by period**			0.279
**Arrhythmia** **No** **Yes**	1489 (92.4%) 122 (7.6%)	67 (98.5%) 1 (1.5%)	0.057
Arrhythmia stratified by period *			**0.032** **OR = 0.19** **(0.01 to 0.89)**
**1st period: No** **Yes**	311 (99%) 3 (1%)	15 (100%) 0	
**2nd period: No** ** Yes**	433 (93.1%) 32 (6.9%)	24 (100%) 0	
**3rd period: No** ** Yes**	400 (90.1%) 44 (9.9%)	17 (94.4%) 1 (5.6%)	
**4th period: No** ** Yes**	345 (88.9%) 43 (11.1%)	11 (100%) 0	
**Diabetes mellitus** **No** **Yes**	1374 (85.3%) 237 (14.7%)	59 (86.8%) 9 (13.2%)	0.736
**Diabetes mellitus stratified by period**			0.908
Smoking habit ** No **20 PY** **40 PY** **Ex**	316 (21.1%) 117 (7.8%) 578 (38.6%) 486 (32.5%) 114 missing	3 (4.6%) 5 (7.7%) 34 (52.3%) 23 (35.4%) 3 missing	**0.009**
Smoking habit stratified by period **			**0.002**
**1st period: No** **20 PY** **40 PY** **Ex**	72 (28.5%) 35 (13.8%) 134 (53.0%) 12 (4.7%) 61 missing	1 (7.7%) 1 (7.7%) 11 (84.6%) 0 2 missing	
**2nd period: No** ** 20 PY** ** 40 PY** ** Ex**	75 (16.5%) 48 (10.5%) 220 (48.4%) 112 (24.6%) 10 missing	0 38 (12.5%) 12 (50%) 9 (37.5%)	
**3rd period: No** ** 20 PY** ** 40 PY** ** Ex**	103 (24.6%) 29 (6.9%) 130 (31%) 157 (37.5%) 25 missing	1 (5.9%) 1 (5.9%) 8 (47.1%) 7 (47.2%) 1 missing	
**4th period: No** ** 20 PY** ** 40 PY** ** Ex**	66 (17.8%) 5 (1.4%) 94 (25.4%) 205 (55.4%) 18 missing	1 (9.1%) 0 3 (27.3%) 7 (63.6%)	
**Lung cancer staging** **0** **I** **II** **IIIA** **IIIB** **IV**	6 (0.4%) 801 (49.7%) 397 (24.6%) 328 (20.4%) 43 (2.7%) 36 (2.2%)	0 30 (44.1%) 15 (22.1%) 19 (27.9%) 4 (5.9%) 0	0.269
**Lung cancer staging stratified by period**			0.450
Preoperative stay (days) *	2.71 (4.92) 1 (1–1) 73 missing	4.81 (9.07) 1 (1–3.5)	**0.016**
Preoperative stay (days) stratified by period **			**0.008**
**1st period**	(n = 314) 9.29 (7.22) 8 (4–13) 36 missing	(n = 15) 14.7 (14.4) 6 (5–20.5)	
**2nd period**	(n = 465) 2.29 (3.91) 1 (1–1) 10 missing	(n = 24) 3.46 (5.13) 1 (1–1.5)	
**3rd period**	(n = 444) 1.03 (0.52) 1 (1–1) 5 missing	(n = 18) 1 (0) 1 (1–1)	
**4th period**	(n = 388) 0.27 (0.71) 0 (0–0) 22 missing	(n = 11) 0.45 (0.52) 0 (0–1)	
**Tumor location** **Intermediate bronchus** **Right main bronchus** **Left main bronchus** **Right lower lobe** **Left lower lobe** **Right middle lobe** **Right upper lobe** **Left upper lobe**	36 (2.2%) 37 (2.3%) 72 (4.5%) 257 (16.0%) 231 (14.3%) 86 (5.3%) 480 (29.8%) 412 (25.6%)	4 (5.9%) 4 (5.9%) 4 (5.9%) 13 (19.1%) 5 (7.3%) 5 (7.3%) 17 (25.0%) 16 (23.5%)	0.094
**Tumor location stratified by period**			0.302
**Resection type** **Bilobectomy** **Lobectomy** **Pneumonectomy: Right, Left** **Segmentectomy**	106 (6.6%) 1063 (66.0%) 109: 37, 72 (6.8%) 333 (20.7%)	7 (10.3%) 43 (63.2%) 8: 4, 4 (11.8%) 10 (14.7%)	0.158
**Resection type stratified by period**			0.339
Local infection ** **No** **Yes**	1565 (97.1%) 46 (2.9%)	60 (88.2%) 8 (11.8%)	**0.001** **OR = 4.54** **(2.05 to 10.03)**
Local infection stratified by period **			**0.002** **OR = 4.45** **(1.99 to 9.96)**
**1st period: No** **Yes**	292 (93%) 22 (7%)	14 (93.3%) 1 (6.7%)	
**2nd period: No** ** Yes**	458 (98.5%) 7 (1.5%)	21 (87.5%) 3 (12.5%)	
**3rd period: No** ** Yes**	433 (97.5%) 11 (2.5%)	14 (77.8%) 4 (22.2%)	
**4th period: No** ** Yes**	382 (98.5%) 6 (1.5%)	11 (100%) 0	
Pneumonia *** **No** **Yes**	1478 (91.7%) 133 (8.3%)	53 (77.9%) 15 (22.1%)	**<0.0001** **OR = 3.15** **(1.73 to 5.73)**
Pneumonia stratified by period ***			**<0.001** **OR = 3.49** **(1.90 to 6.41)**
**1st period: No** **Yes**	297 (94.6%) 17 (5.4%)	14 (93.3%) 1 (6.7%)	
**2nd period: No** ** Yes**	439 (94.4%) 26 (5.6%)	18 (75%) 6 (25%)	
**3rd period: No** ** Yes**	405 (91.2%) 39 (8.8%)	14 (77.8%) 4 (22.2%)	
**4th period: No** ** Yes**	337 (86.9%) 51 (13.1%)	7 (63.6%) 4 (36.4%)	
Empyema *** **No** **Yes**	1583 (98.3%) 28 (1.7%)	42 (61.8%) 26 (38.2%)	**<0.0001** **OR = 35.00** **(18.91 to 64.77)**
Empyema stratified by period ***			**<0.0001** **OR = 34.95** **(18.60 to 65.65)**
**1st period: No** **Yes**	303 (96.5%) 11 (3.5%)	6 (40%) 9 (60%)	
**2nd period: No** ** Yes**	458 (98.5%) 7 (1.5%)	15 (62.5%) 9 (37.5%)	
**3rd period: No** ** Yes**	437 (98.4%) 7 (1.6%)	12 (66.7%) 6 (33.3%)	
**4th period: No** ** Yes**	385 (99.2%) 3 (0.8%)	9 (81.8%) 2 (18.2%)	
**Atelectasis** **No** **Yes**	1478 (91.7%) 133 (8.3%)	58 (85.3%) 10 (14.7%)	0.062
**Atelectasis stratified by period**			0.111
Hemothorax ** **No** **Yes**	1568 (97.3%) 43 (2.7%)	61 (89.7%) 7 (10.3%)	**0.003** **OR = 4.18** **(1.81 to 9.68)**
Hemothorax stratified by period **			**0.004** **OR = 4.26** **(1.83 to 9.90)**
**1st period: No** **Yes**	304 (96.8%) 10 (3.2%)	12 (80%) 3 (20%)	
**2nd period: No** ** Yes**	458 (98.5%) 7 (1.5%)	23 (95.8%) 1 (4.2%)	
**3rd period: No** **Yes**	427 (96.2%) 17 (3.8%)	15 (83.3%) 3 (16.7%)	
**4th period: No** ** Yes**	379 (97.7%) 9 (2.3%)	11 (100%) 0	
**Pulmonary thromboembolism** **No** **Yes**	1598 (99.2%) 13 (0.8%)	66 (97.1%) 2 (2.9%)	0.121
**Pulmonary thromboembolism stratified by period**			0.163
**Perioperative AMI** **No** **Yes**	1597 (99.1%) 14 (0.8%)	67 (98.5%) 1 (1.5%)	0.464
**Perioperative AMI stratified by period**			0.279
**Chylothorax** **No** **Yes**	1606 (99.7%) 5 (0.3%)	68 (100%) 0	1
**Chylothorax stratified by period**			0.540
Reintervention *** **No** **Yes**	1568 (97.3%) 43 (2.7%)	35 (51.5%) 33 (48.5%)	**<0.0001** **OR = 34.38** **(19.56 to 60.42)**
Reintervention stratified by period ***			**<0.0001** **OR = 37.29** **(20.66 to 67.31)**
**1st period: No** **Yes**	297 (94.6%) 17 (5.4%)	5 (33.3%) 10 (66.7%)	
**2nd period: No** ** Yes**	456 (98.1%) 9 (1.9%)	17 (70.8%) 7 (29.2%)	
**3rd period: No** ** Yes**	430 (96.8%) 14 (3.2%)	8 (44.4%) 10 (55.6%)	
**4th period: No** ** Yes**	385 (99.2%) 3 (0.8%)	5 (45.5%) 6 (54.5%)	
Operative mortality * **No** **Yes**	1611 (100%) 0	67 (98.5%) 1 (1.5%)	**0.041** **OR = 71.62** **(2.89 to 1774.48)**
Operative mortality stratified by period *			**0.022** **OR = 56.86** **(6.14 to 241.64)**
**1st period: No** **Yes**	314 (100%) 0	15 (100%) 0	
**2nd period: No** ** Yes**	465 (100%) 0	24 (100%) 0	
**3rd period: No** ** Yes**	444 (100%) 0	18 (100%) 0	
**4th period: No** **Yes**	388 (100%) 0	10 (90.9%) 1 (9.1%)	
Postoperative mortality ** **No** **Yes**	1560 (96.8%) 51 (3.2%)	60 (88.2%) 8 (11.8%)	**0.002** **OR = 4.08** **(1.85 to 8.97)**
Postoperative mortality stratified by period **			**0.004** **OR = 3.78** **(1.71 to 8.35)**
**1st period: No** **Yes**	299 (95.2%) 15 (4.8%)	14 (93.3%) 1 (6.7%)	
**2nd period: No** ** Yes**	442 (95.1%) 23 (4.9%)	23 (95.8%) 1 (4.2%)	
**3rd period: No** ** Yes**	434 (97.7%) 10 (2.3%)	15 (83.3%) 3 (16.7%)	
**4th period: No** **Yes**	385 (99.2%) 3 (0.8%)	8 (72.7%) 3 (27.3%)	

**Table 4:** Multivariable logistic regression model for the entire target population with fistula as the dependent outcome. There were 149 patients deleted due to missing values in some of the variables introduced in the stepwise procedure. Effective sample size n = 1530. Stepwise procedure carried out from the following identified risk factors in the univariate analyses, excluding those related to other complications: sex, COPD, arterial hypertension, vasculopathy, arrythmia, diabetes mellitus, smoking habit, lung cancer staging, preoperative stay, resection type, and HNC. The final fit, which retained sex, HNC, COPD and preoperative stay as independent variables at a significant level of 0.05, yields an optimistic AUC = 0.732 (95% CI: 0.674 to 0.791).

Parameter	Adjusted OR	Z Statistic	*p*-Value	95% CI (OR)		VIF
**Intercept**	0.025	−15.141	<0.0001	0.016	0.041	
**Sex (Female)**	0.173	−2.419	0.0155	0.042	0.716	1.011
**HNC (Yes)**	3.654	3.616	0.0003	1.810	7.374	1.014
**COPD (Yes)**	2.122	2.780	0.0054	1.248	3.607	1.084
**Preoperative stay (days)**	1.065	3.637	0.0003	1.030	1.102	1.093

**Table 5:** Multivariable logistic regression model for the entire target population with BPF presence as the dependent outcome. There are 73 patients deleted due to missing values in preoperative stay. Effective sample size n = 1606. This fit yields an optimistic AUC = 0.723 (95% CI: 0.665 to 0.781).

Parameter	Adjusted OR	Z Statistic	*p*-Value	95% CI (OR)		VIF
**Intercept**	0.027	−15.683	<0.0001	0.018	0.043	
**Sex (Female)**	0.161	−2.523	0.0116	0.039	0.665	1.010
**HNC (Yes)**	3.562	3.569	0.0004	1.773	7.156	1.013
**COPD (Yes)**	1.947	2.550	0.0108	1.167	3.251	1.073
**Preoperative stay (days)**	1.060	3.414	0.0006	1.025	1.096	1.082

**Table 6:** Multivariable logistic regression model for the entire target population with BPF as the dependent outcome. The missing values of preoperative stay have been imputed with the median value (1 day). Effective sample size n = 1679. This fit yields an optimistic AUC = 0.729 (95% CI: 0.671–0.786).

Parameter	Adjusted OR	Z Statistic	*p*-Value	95% CI (OR)		VIF
**Intercept**	0.025	−16.115	<0.0001	0.016	0.040	
**Sex (Female)**	0.164	−2.494	0.0126	0.040	0.679	1.010
**HNC (Yes)**	3.678	3.661	0.0003	1.831	7.388	1.013
**COPD (Yes)**	2.047	2.747	0.0060	1.228	3.412	1.068
**Preoperative stay (days)**	1.063	3.605	0.0003	1.028	1.099	1.077

**Table 7:** Multivariable logistic regression model of Table 4 with period of surgery included as another independent variable. Effective sample size n = 1530. This fit yields an optimistic AUC = 0.729 (95% CI: 0.667 to 0.791).

Parameter	Adjusted OR	Z Statistic	*p*-Value	95% CI (OR)		VIF
**Intercept**	0.016	−9.048	<0.0001	0.006	0.039	
**Sex (Female)**	0.171	−2.410	0.0159	0.041	0.719	1.030
**HNC (Yes)**	3.789	3.677	0.0002	1.863	7.709	1.032
**COPD (Yes)**	2.042	2.599	0.0093	1.192	3.499	1.112
**Preoperative stay (days)**	1.083	3.613	0.0003	1.037	1.130	1.802
**Period of surgery (2)**	1.861	1.412	0.1581	0.786	4.409	1.866
**Period of surgery (3)**	1.564	0.906	0.3649	0.595	4.113	
**Period of surgery (4)**	1.588	0.865	0.3871	0.557	4.532	

**Table 8:** Multivariable logistic regression model of Table 4 with smoking habit included as another independent variable. Effective sample size n = 1530. This fit yields an optimistic AUC = 0.746 (95% CI: 0.688 to 0.805).

Parameter	Adjusted OR	Z Statistic	*p*-Value	95% CI (OR)		VIF
**Intercept**	0.009	−7.517	<0.0001	0.003	0.031	
**Sex (Female)**	0.230	−1.998	0.0458	0.055	0.973	1.032
**HNC (Yes)**	3.427	3.397	0.0007	1.684	6.976	1.036
**COPD (Yes)**	1.921	2.389	0.0169	1.124	3.283	1.101
**Preoperative stay (days)**	1.069	3.591	0.0003	1.031	1.108	1.207
**Smoking habit (20 PY)**	2.525	1.227	0.2200	0.575	11.092	1.185
**Smoking habit (40 PY)**	3.322	1.918	0.0551	0.974	11.331	
**Smoking habit (Ex)**	3.276	1.833	0.0669	0.921	11.654	

## Data Availability

The data presented in this study are available in this article.

## Abbreviations

HNC	Head and neck cancer
LC	Lung cancer
COPD	Chronic obstructive pulmonary disease
AMI	Acute myocardial infarction
PYI	Pack-year index
PY	Pack-year
BPF	Bronchopleural fistula

## Appendix A

**Table A1:** Number of patients included in every time period covered by this study.

Time Period	n	%
1989–1999	329	19.6
1990–2009	489	29.1
2010–2019	462	27.5
2020–2024	399	23.8

**Table A2:** Second primary lung cancer location.

Lung Cancer Location	n	%
Right upper lobe	497	29.6
Right middle lobe	91	5.4
Right lower lobe	270	16.1
Intermediate bronchus	40	2.4
Right main bronchus	41	2.4
Left upper lobe	428	25.5
Left lower lobe	236	14.1
Left main bronchus	76	4.5

**Table A3:** Second primary lung cancer histologic type.

LC Histologic Type	n	%
Squamous-cell carcinoma	758	45.1
Adenocarcinoma	728	43.4
Large-cell lung carcinoma	67	4.0
Small-cell lung carcinoma	20	1.2
Neuroendocrine tumor	84	5.0
Other	22	1.3

**Table A4:** Second primary lung cancer resection type.

LC Resection Type	N	%
Lobectomy	1106	65.9
Bilobectomy	113	6.7
Segmentectomy	343	20.4
Pneumonectomy	117	7
- Right	41	2.5
- Left	76	4.5

**Table A5:** Previous HNC location.

HNC Location	n	%
Pharynx	7	8.05
Larynx	56	64.4
Tongue	11	12.6
Oral cavity	7	8.05
Other	6	6.9

**Table A6:** Previous HNC histological type.

HNC Histological Type	N 87
Squamous-cell carcinoma	81
Other	6

**Table A7:** Previous HNC treatment.

HNC Treatment	n	%
Surgery	33	37.9
RT	1	1.2
Surgery + RT	32	36.8
QT + RT	11	12.6
Surgery + QT	4	4.6
Surgery + QT + RT	2	2.3
Endoscopic laser therapy	4	4.6

**Table A8:** Time interval from primary HNC to second primary LC (in months).

Time Interval from HNC to LC	Mean (SD): 70.0 (57.1)	Median (Q1, Q3): 61.0 (20.5, 102.5)
≤1 year	14	16.1
1–5 years	28	32.2
5–10 years	30	34.5
>10 years	15	17.2
